# A spaced-repetition approach to enhance medical student learning and engagement in medical pharmacology

**DOI:** 10.1186/s12909-022-03324-8

**Published:** 2022-05-02

**Authors:** Dylan Jape, Jessie Zhou, Shane Bullock

**Affiliations:** Monash Rural Health, Medicine, Nursing & Health Sciences, Monash University, Northways Road, Churchill, VIC 3842 Australia

**Keywords:** Medical pharmacology, Spaced-repetition, Flashcards, Medical education

## Abstract

**Background:**

Pharmacology is a cornerstone of medical education as it underlies safe prescribing practices. However, medical students have reported unease regarding their perceived proficiency in medical pharmacology. Despite the significant impetus to improve student outcomes, there is little analysis available of the techniques used by medical students to learn, retain and apply medical pharmacology knowledge.

**Methods:**

A mixed-methods, student-focused approach was conducted to design and evaluate specific resources developed to address gaps in pharmacology education. This methodology comprised an anonymised scoping survey, followed by semi-structured focus group interviews. We then developed a flashcard resource as an intervention to support long-term revision for academic and clinical success. This resource was released to a cohort of 100 graduate entry preclinical medical students who were invited at the end of year to evaluate the intervention via a subsequent anonymous survey.

**Results:**

The scoping survey received 103 complete responses. Surveys and focus group interviews revealed that only 50% of students engage in ongoing revision. Amongst our cohort, we identified that the evidence-based technique of spaced-repetition was particularly well regarded. Hence, we developed and evaluated a bespoke resource utilising Anki™, an open-source, spaced-repetition flashcard program. A total of 1208 flashcards spanning 156 distinct classes of drugs with supplementary summary tables, diagrams and explanatory video and summary guides were created. Designed as a strategic revision tool to reinforce learning, evaluation showed students greatly appreciated the “comprehensive” and “well formatted” Anki™ resource that supported existing teaching modalities, with a global rating of 3.8 out of 5.

**Conclusions:**

Strategic and personalised resources for medical pharmacology education that assist with in-semester revision and long-term retention are highly valued amongst students for examination preparation and preparedness for practice. Collectively, these results reflect a novel approach to identifying and addressing weaknesses in existing learning resources in a manner that is inclusive of, and acceptable to, medical students.

**Supplementary Information:**

The online version contains supplementary material available at 10.1186/s12909-022-03324-8.

## Background

Competence in medical pharmacology is a crucial outcome in medical education as the discipline provides the rationale for therapeutic interventions and safe prescribing. However, significant challenges face medical students, such as developing expertise associated with the vast and ever-expanding array of medications available on the market, each with unique clinical considerations and interactions [1]. Given this, it is unsurprising that many medical students express concerns regarding competence in medical pharmacology and prescribing, with only 39% of Australian medical graduates perceiving that they were adequately or well prepared in medical pharmacology [2, 3].

Thus, there is significant interest in educational reforms to improve student outcomes in medical pharmacology. However, it is notable that most approaches have been top-down in nature, focusing on systematic changes in curriculum, teaching and staffing [4]. Hence, there is a gap in the understanding of the approaches and techniques used by medical students to study medical pharmacology, which could form the basis for targeted interventions to improve learning [5–8].

Of interest is how students cope with fast-paced integrated medical school curricula and whether support can be provided to encourage more efficient approaches to study [9]. One technique of interest is spaced-repetition of flashcards, such as via the open-source and cross-platform digital flashcard program Anki™, which is known to improve student outcomes [10–13]. This method of learning involves self-testing on flashcards that assess specific items of knowledge, which are subsequently self-rated for difficulty. The program subsequently sets intervals for re-assessment, depending upon prior difficulty ratings, establishing a personalised schedule for revision.

**Fig. 1:**
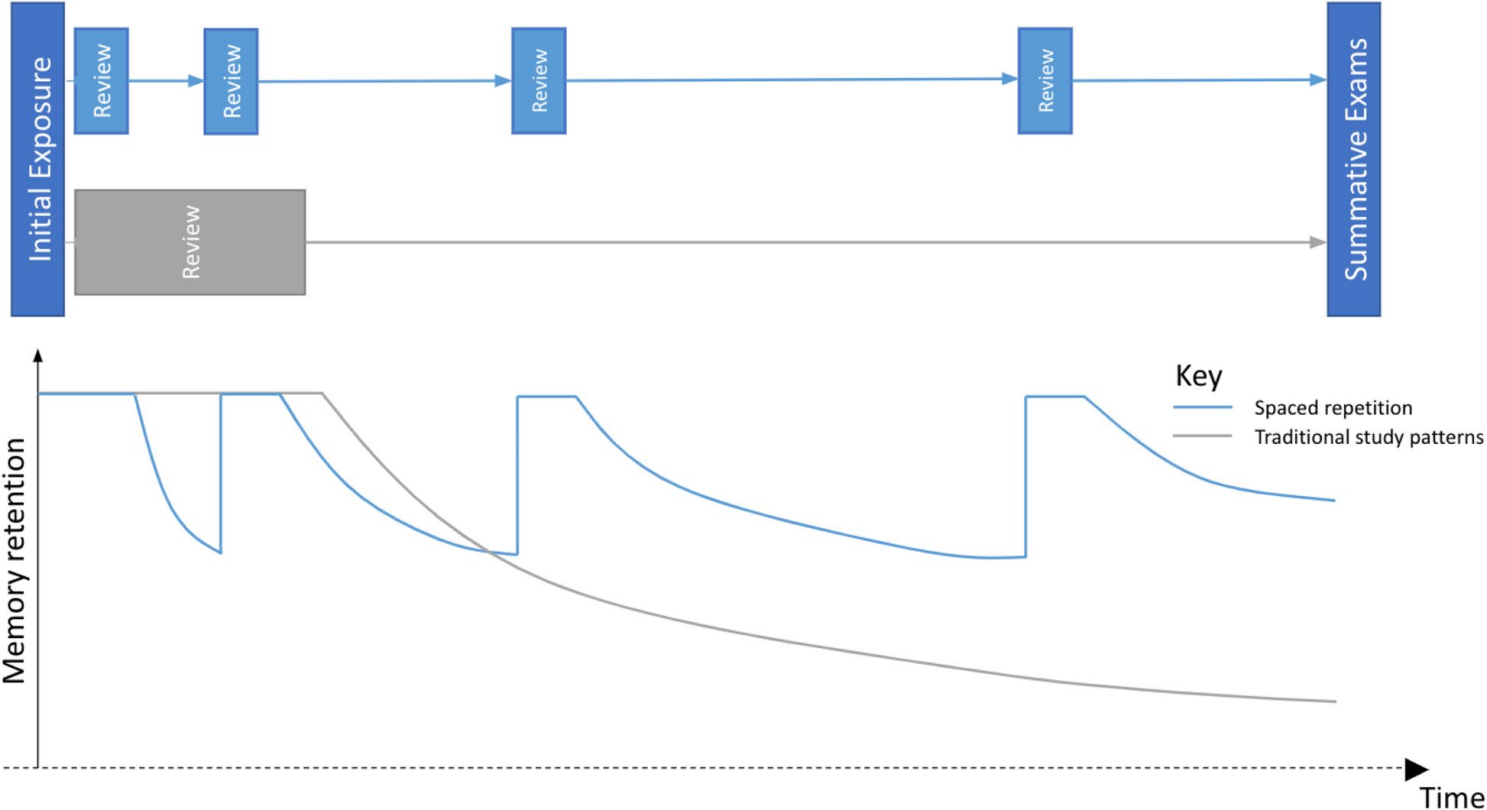
Comparison of exposures to a knowledge with spaced-repetition and traditional techniques. This diagram illustrates the early focus of traditional learning techniques on amassing practice, which creates an initially strong memory that ultimately decays without further revision. By comparison, spaced-repetition is known to effectively distribute meaningful practice across time to reinforce learning and slow the decay of memory, promoting long-term retention [20]. Individual flashcards are personally scheduled based upon user ratings of difficulty, with the interval between revision instances reduced for more difficult flashcards, improving the retention of knowledge. Conversely, easier flashcards are given longer intervals to both increase time efficiency and ensure meaningful challenge in revision

Spaced-repetition carries a number of distinct advantages when considered in the context of supporting medical student education [14]. As a form of flashcard revision, spaced-repetition engages students in self-assessment with immediate feedback to take advantage of the testing effect, where testing knowledge produces superior learning outcomes compared to studying the knowledge itself [15–17]. Such a resource is envisioned to support traditional modes of teaching, the flashcards are all formatted to involve free recall of items of knowledge, which has significant advantages over more passive methods of learning such as re-presentation and even multiple-choice questions [18–20]. Spaced-repetition may enhance time efficiency of revision as the program prioritises revision of poorly understood items and minimises time wasted revising well understood items, optimising the degree of difficulty required for effective revision (Fig. 1) [20]. In being regularly reviewed by members of faculty, the resource concentrates valuable information into a single location for students to readily access.

In this study, we examine the perceptions and approaches of medical students to learning medical pharmacology, with a focus on identifying and targeting weaknesses through the development of supportive educational resources. In this way, we aim to contribute to a greater understanding of both student perspectives towards learning and possible actionable interventions to improve outcomes.

## Methods

Students involved in this study were enrolled in either direct entry (also known as undergraduate entry) or graduate entry Doctor of Medicine (MD) streams delivered by an Australian university. The direct entry pathway is a five-year program where students enter the course directly from secondary school, while the graduate entry course is of four years, where students enter after completion of an appropriate Bachelor degree (or higher). Each year level has approximately 350 direct entry students and 100 graduate entry students. Currently enrolled medical students at all year levels were invited to participate via advertisements on student notice boards. A total number of 2,300 students were eligible to participate in the survey.

The medical school’s pharmacology curriculum at this university is structured to build knowledge and skills over the whole course. In the preclinical phase of the course, students become familiarised with basic pharmacology principles and are introduced with a strong clinical context to the actions, adverse effects and major clinical considerations associated with the major therapeutic drug groups. In the first and second clinical years, medical pharmacology is focused on appropriate choices of drugs and the adverse effects of specific agents as they relate to clinical encounters with patients. In the final clinical year, the development of prescribing knowledge and skills is important.

Ethics approval was sought from the university’s human ethics committee prior to commencement (MURHEC number: 2020–22814-44162). All methods were carried out in accordance with relevant guidelines and regulations. We estimated that the time commitment required for the survey would not exceed 20 min. Students were not given a payment in exchange for their time; rather, as stated in the Project Explanatory Statement, their involvement may help improve the course for both themselves and future medical students.

**Fig. 2:**
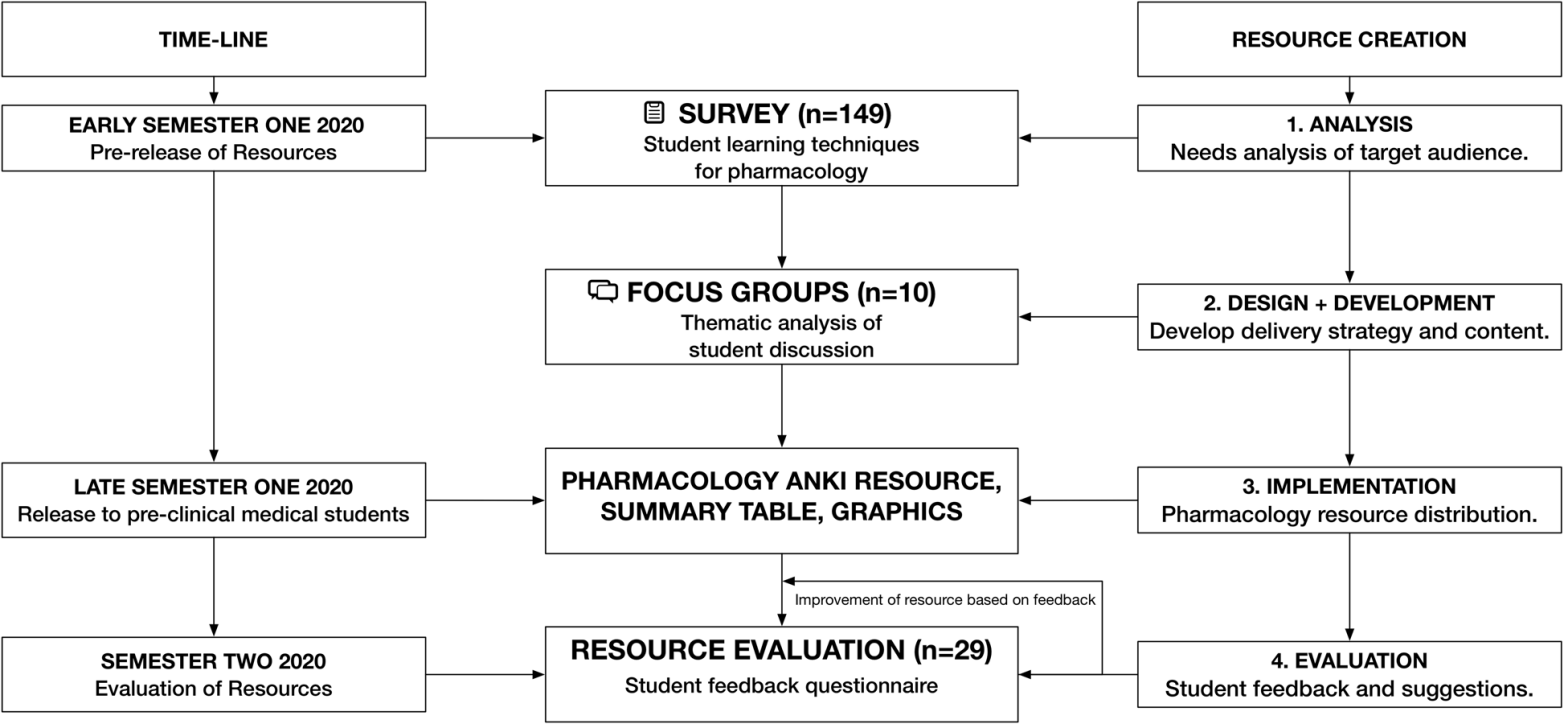
Flow-chart of resource development and evaluation. This diagram highlights the stepwise iterative process of resource creation. The initial scoping survey of medical students guided the design, development and implementation of bespoke medical pharmacology resources with evaluation and feedback processes for quality assurance and continual improvement

To better understand students' approaches to medical pharmacology education, they were invited to complete an anonymised scoping survey. An invitation to participate in a subsequent structured focus group interview was provided at the end of the survey, with a goal of further elucidating survey findings and to demonstrate and refine specific resources developed. These resources were only released to a cohort of graduate entry preclinical medical students who were invited at the end of year to evaluate the intervention via an anonymous follow-up survey (Fig. 2).

### Initial scoping survey design and data analysis

To better understand the perceptions and approaches of medical students with respect to medical pharmacology education, an initial scoping survey was made available to current students enrolled in the MD program. The survey primarily consisted of 5-point Likert scales and free text items.

The survey consisted of two distinct sections. The first section was targeted at identifying key demographic characteristics of the survey participants such as age, sex, year level, entry stream and whether the students were domestic or international. The second section of the survey was designed to identify the resources and techniques utilised by students to learn medical pharmacology, with a Likert scale utilised to gauge use of common study techniques, with free text options for unlisted techniques (Appendix 1). These characteristics were assessed with regard to three distinct time periods of learning; upon first exposure to a topic, as ongoing revision and as revision immediately prior to summative assessments**.** Statistical analysis of the data was performed to identify significant associations via cross-tabulation and Student’s t-test using MS Excel.

### Focus group design and data analysis

Survey respondents were also invited to participate in focus groups to further elicit qualitative information on survey findings and to evaluate several prospective study resources. Two rounds of focus group interviews with medical students were performed, with a total of ten student participants.

The student participants were coded as follows:

-
  - Focus Group A: 4 students (A1, A2, A3, A4)
-
  - Focus Group B: 6 students (B1, B2, B3, B4, B5, B6)

The design and data analysis of this study was informed by a grounded theory approach, where an understanding of what happened is grounded in the data derived from the shared experiences of the participants and researchers [21]. The methodology used in this study is as per that described by Watling & Lingard [22].

### Flashcard spaced-repetition resource design and implementation

Evaluation of the primary survey identified Anki™ flashcards, supported by text based summaries and diagrams, as suitable resources for a potential intervention to support medical pharmacology learning. The Anki™ software carries numerous advantages as an open-source application that is free of charge on several platforms, including PC, Mac and Android (also available on iPhone, but as a paid application). These resources were developed for initial evaluation within focus group interviews and later, with a cohort of preclinical graduate entry medical students.

**Fig. 3:**
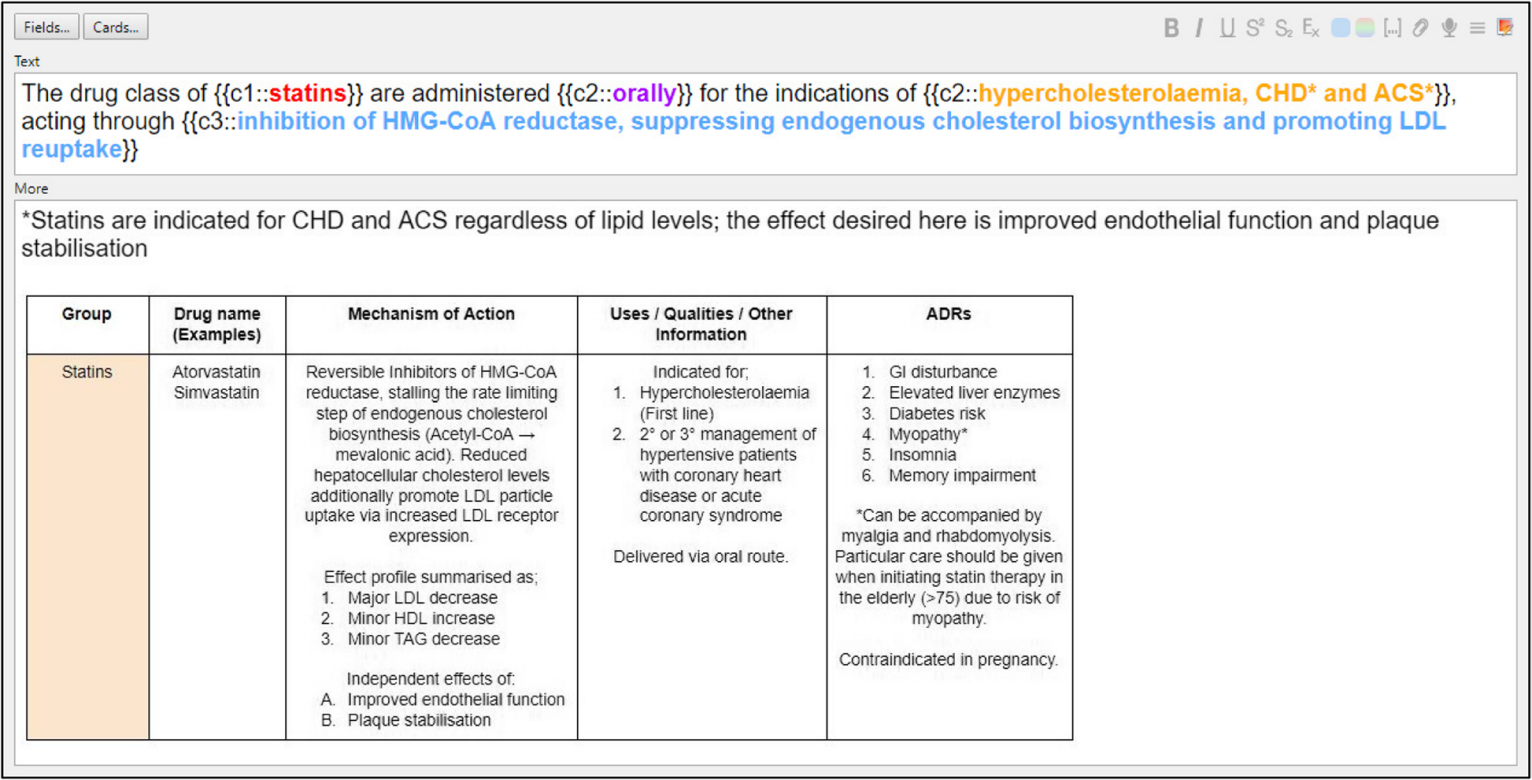
Example design of Anki™ medical pharmacology flashcards. This view shows the back-end editing view of Anki™ flashcards, demonstrating the use of three distinct clozes, here represented in different colours, within a single note to produce three distinct flashcards that assess different items of knowledge. When reviewing, the students will be given the text section with one cloze blank, which tests the student’s ability to freely recall the missing information. Once answered, the program will flip the card to show a model answer and the more information section of the flashcard

Flashcards were designed as cloze sentences, which are sentences with missing words testing a student’s recall and understanding of an item of content (Fig. 3). Students were then given the opportunity to check their answer and rate the card difficulty, feeding into the spaced-repetition algorithm of Anki™ to personally schedule future revision based upon need. Anki™ flashcards were created to cover 15 core curriculum topics, spanning 156 distinct classes of drugs, resulting in a total of 1208 flashcards, corresponding to the scope of the pre-clinical medical pharmacology program. Flashcards assessed a diverse range of curriculum material, including basic knowledge such as drug names, mechanisms, indications and adverse drug reactions to more complex knowledge such as clinical practice guidelines. The Anki™ decks were student-initiated; created by the student author (DJ), which were then reviewed for relevance by the pharmacology lecturer (SB) delivering the content and validated by the course graduate and now junior doctor (JZ), who was a qualified pharmacist before enrolling in the medicine course. On average, each core curriculum flashcard deck is equivalent to 1 h of lecture material and took 3 h to create.

Traditional text and diagrammatic summaries were also developed to complement the Anki™ resource, allowing students to review the details of the broader drug class and clinical pharmacology topics both within and independent of the Anki™ program.

### Intervention follow-up survey design and data analysis

To evaluate the effectiveness of the intervention within the population of preclinical graduate entry medical students, a follow-up survey was distributed via advertisements on student forums and noticeboards. Student perceptions of the medical pharmacology resources were elicited using 5-point Likert scales and free text items, with data subsequently analysed using MS Excel.

## Results

### Initial scoping survey findings

The survey received 103 complete responses from the eligible population of approximately 2,300 enrolled students across all years of the medical course. This represents a response rate of 4.5%. The demographic profile of the respondents was a median age of 22 years and 69% female. Fifty-six percent of participants were enrolled in the direct stream, with being 81% domestic students. Twenty-six per cent of respondents were in their preclinical years, 44% in their first clinical year, 11% in their second clinical year and 20% in their final year of the course.

### Student learning preference and approaches

With respect to three distinct stages of learning engagement (first learning a new topic, revision during semester and revision prior to major assessments), most participants reported significant engagement in dedicated medical pharmacology study to learn new material (95%) and to revise prior to exams (80%). However, only 50% of respondents reported specific medical pharmacology revision throughout the teaching year.

When participants were asked about their usage of several learning techniques of interest (Table 1), students showed a significant preference towards class activities (63%), text-based notes (84%) and faculty question banks (60%). Relative to the overall level of engagement with studying during semester and prior to exams, students largely reviewed or repeated techniques utilised to learn medical pharmacology.

**Table 1:** Comparison of medical pharmacology study technique popularity amongst students. This was assessed via a 5-point Likert scale, with the proportion of strong or very strong engagement responses reported

Technique	First Exposure Learning (%)	In-semester revision (%)	Exam revision (%)
Class activities or resources	63	41	56
Personal text-based notes	84	40	78
Concept maps	23	18	26
Flow chart	29	18	27
Diagrams	46	23	42
Faculty question banks	60	30	58
Flashcards	35	30	63

One notable exception was in the use of flashcards, which had a net increase in usage as in-semester review (30%) and exam revision (63%) as compared to first exposure learning (35%), despite the lower levels of student engagement at the later time points. Amongst the distinct forms of flashcard revision examined, digital flashcards with spaced-repetition scheduling (e.g. Anki™) were the most popular means of study reported.

### Focus group results

The focus group participants were 70% female, 70% direct entry medical students and 50% preclinical students. The two overarching categories that emerged from the focus groups were ‘Medical pharmacology learning’ and ‘Utility of flashcards in revision’.

#### Category 1: medical pharmacology learning

In relation to medical pharmacology learning, two themes were quite apparent: the student experience and barriers to learning. In general, medical pharmacology teaching was well received; students particularly enjoyed the clinically integrated case-based approach to learning.

*“I feel like we have a good amount of clinical cases given to us. Because it is definitely easier to relate what you learn when you learn about someone who is going through that condition.”* Student B4 (preclinical, direct entry)

However, there are barriers to effective medical pharmacology learning as identified by students. The two most common were time management and difficulty with long-term retention.

*“Some way of keeping students somewhat accountable for not trying to cram everything at the end of the year.”* Student B3 (preclinical, direct entry)

*“I think there should be more emphasis on learning how to learn… You could be studying a lot of hours but you might not be studying effectively.”* Student B5 (preclinical, direct entry)

*“Actually during exams I actually knew all my Pharmacology really well, but if you were to ask me anything right now ...I wouldn’t be able to answer anything. It is just not sticking in my long-term memory.”* Student B5 (preclinical, direct entry)

### Category 2: flashcards as revision tools

For this category, the two key themes were student engagement and limitations of use. Student engagement with flashcards was generally positive with perceptions of time efficiency and a relatively active form of learning.

*“I just find flash cards are a much more active way to study, so in terms of efficiency, you can learn and retain the material in a shorter amount of time, which is what we’re all looking for.” Student A3 (preclinical, graduate entry).*

*“I was excited by it, because I've used Anki™ all through my biomed degree and found it a really good way to study actively, and I also really like the structure of having cards to organise my revision.” Student A3 (preclinical, graduate entry).*

Some students also identified flashcards as facilitating longer-term retention of knowledge.

*“That long-term…knowledge would be good … and that’s what Anki™’s there for … the way that you’ve pitched it is also quite appropriate for what it should be used for.” Student A1 (clinical, graduate entry).*

Clear and concise study resources are crucial for student uptake, understanding and engagement. The format of Anki™ Flashcards permits only relevant key points to be presented, making it an attractive learning tool for students.

*“Flash cards would be fantastic because it is very concise …. [with] an actual real-world example that would be so much better.” Student B2 (clinical, direct entry).*

An important limitation of use of the Anki™ resource was that it was perceived as “intimidating”, particularly for students who have not previously utilised flashcards as a study resource.

*“I talked to a couple of people who have started using it; but I think they found it quite difficult to go through without going through slides and pre-learning beforehand.” Student A3 (preclinical, graduate entry).*

*“There are so many cards it is a bit intimidating to try and learn them.” Student B4 (clinical, direct entry).*

The introduction of a guide and incorporating flashcards into the study workflow as a post lecture revision tool was seen as useful strategies to enable students to fully appreciate the utility of the Anki™ resource*.*

*“Write a manual …how to use each thing, like step by step? …Written down; so people can access it at any time.” Student A2 (preclinical, graduate entry).*

### Intervention follow-up survey findings

**Fig. 4:**
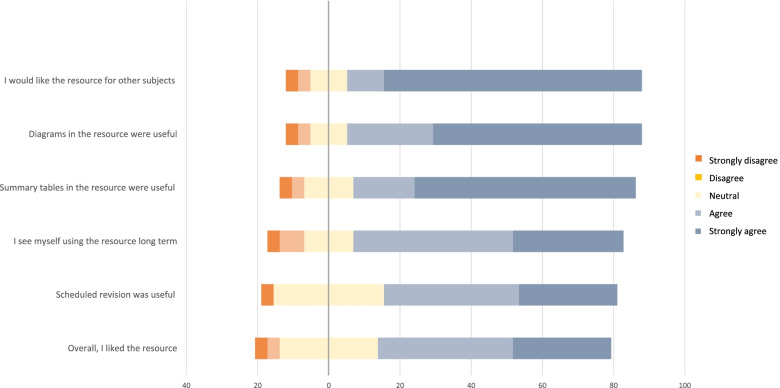
Likert scale rating of student-guided learning interventions. The Figure demonstrates the proportion of different student responses to a 5-point Likert scale evaluating the Anki™ resource developed and implemented from insights gained via the primary survey

The follow-up survey of preclinical graduate entry students given access to the resource received a response rate of 29%. Reported usage of the Anki™ resource was high at 83%, with 52% of students reporting engagement with the resource to complete all scheduled revision and 31% reporting engagement with the Anki™ resource as a traditional question bank for episodic revision. Overall, the Anki™ resource was well received, with an average rating of 3.8 out of 5. Furthermore, 66% of participants positively received the scheduling of revision by Anki™ and 76% positively rated the long-term viability of Anki™ as a study technique. Notably, 83% of students positively perceived the benefits of Anki™ as a study technique for subjects other than medical pharmacology. Furthermore, the accompanying summary tables and diagrammatic representations within the resource package were positively received by 79% and 83% of students respectively (Fig. 4).

When participants in the intervention follow-up survey were asked about the strengths of the Anki™ medical pharmacology resource and how it could be improved, they gave similar responses to focus group interviewees that the resource was considered a *“great”* and *“comprehensive” “study system”* for *“continuous revision”* of key medical pharmacology concepts. Students highlighted that the resource is not only immediately valuable for their studies for revision and assessment preparation, but long-term knowledge retention in clinical years and future practice as an “*accurate resource to refer back to”* that *“highlights key concepts that we should know not for just this year, but for later years.”*

However, in creating a comprehensive and detailed resource for preclinical students that incorporates clinically-applied pharmacology, 45% of students expressed sentiments that *“the sheer volume of some decks felt overwhelming”* and that some decks included information *“outside the scope”* of the preclinical pharmacology syllabus. Integration of the Anki™ resource with case examples and links to useful resources and videos for certain drugs was another suggestion for further development of the resource. Additionally, requests for *“more visual schematics”* to be incorporated in the flashcards re-emphasised feedback from the initial scoping survey and focus group discussions.

## Discussion

Revision and self-testing are critical acts in the process of learning, aiding students in consolidation of their knowledge and building confidence in practice [10]. With the demands of modern medical school curricula, students are placed under significant pressure to remain up to date with their course content and thus, have significant time barriers impeding revision. Here, we found that whilst 95% of participants engaged in dedicated medical pharmacology study to learn new content and 80% revised prior to exams, only 50% revised during the semester. Qualitatively, participants reported that such revision was often superficially motivated by summative assessment deadlines, rather than for longer-term competency. This is consistent with existing studies that suggest students often adopt less effective shallow strategies for learning such as rereading, highlighting and cramming [23, 24]. Therefore, interventions focusing on engaging medical students in ongoing revision and active learning provides clear benefits for student outcomes [25].

Beyond simply encouraging ongoing revision, our examination of the student approaches to learning yielded significant insights. We found that students are acutely aware of the significant learning expectations and time pressures placed upon them at an early stage in their medical school journeys, and thus valued time efficiency and evidenced based strategies for studying. Many were well aware of techniques such as spaced-repetition, but found significant barriers to entry in learning how to use the software and creating their own flashcards in addition to existing learning and time commitments. Unfortunately, this appears to have contributed to an undesirable situation where students that are struggling with time management are the least equipped to access and engage with more efficient study techniques.

Based upon these findings, we postulated that development and implementation of a comprehensive Anki™ medical pharmacology resource, based upon the medical pharmacology curriculum would bear several advantages. As a developed resource on an open source program, such an intervention would lower the major barrier to entry for students to engage in curriculum-specific pharmacology revision that is time-efficient and freely accessible [26]. Such a resource could encourage more effective revision involving principles such as retrieval practice and free recall [24], as well as the option to study with spaced-repetition scheduling that maximises time spent on challenging concepts to improve outcomes [13, 27]. The digital nature of the flashcards allows for ease of editing, collaboration and access, thereby ensuring the sustainability of the resource in the long-term [14].

**Fig. 5:**
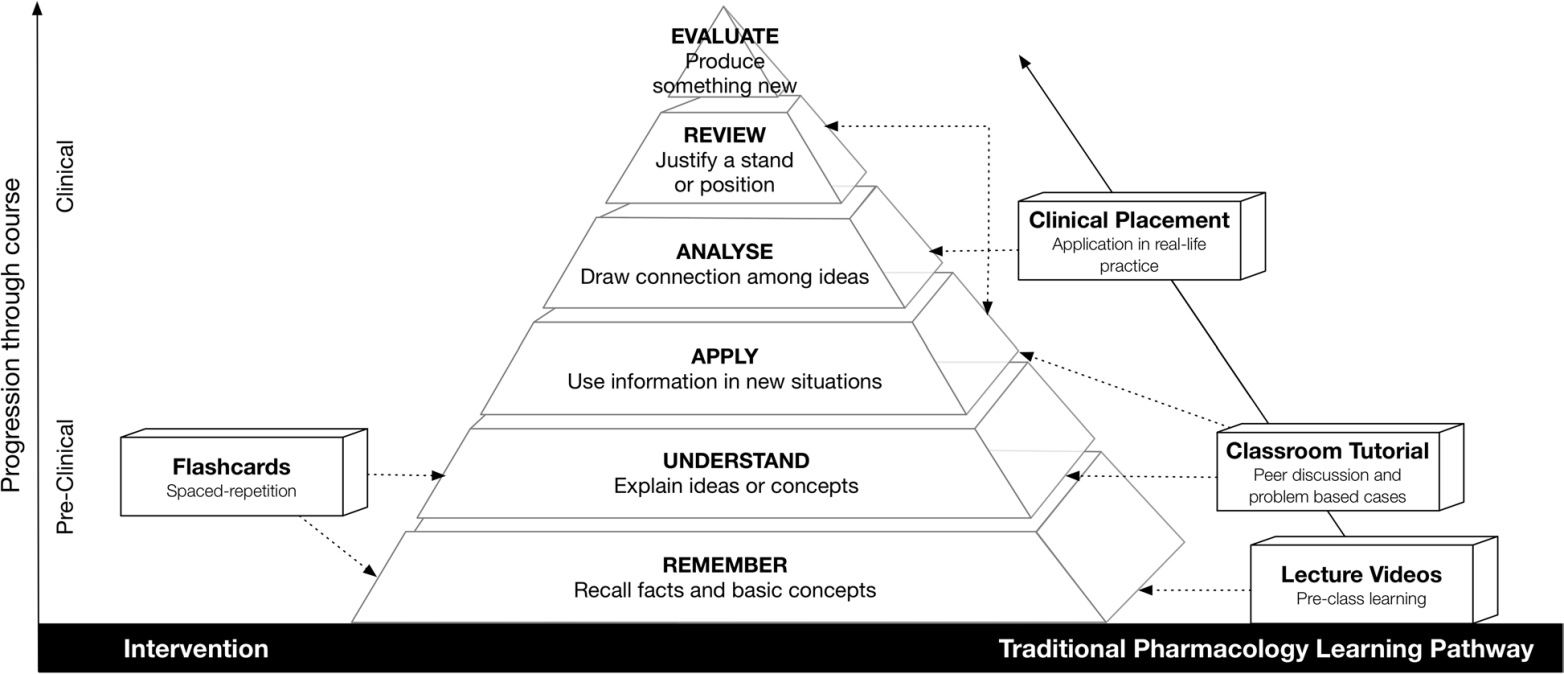
Incorporation of Anki™ Spaced-repetition flashcards into medical pharmacology teaching articulating with Bloom’s Taxonomy framework for learning. The Figure demonstrates how resources such as our flashcard intervention that assists with remembering and understanding of materials complements traditional methods of teaching and helps to develop further learning as students’ progress throughout the course

The extensive Anki™ flashcard deck was subsequently incorporated into the medical pharmacology program as an intervention with completed decks of flashcards released to the participating students following traditional classes as a form of ongoing revision in accordance with Bloom’s Taxonomy (Fig. 5) [28]. Incorporation of Anki™ into the pedagogy, may provide reassurance for teachers that students engaging with the spaced-repetition tool are periodically self-testing their memory and understanding of delivered material, reinforcing recall of foundational knowledge and checking understanding of concepts. A key advantage of this resource is the feedback provided to students during self-testing. This not only identifies difficult concepts, but also provides text and diagrammatic references within the flashcards to allow for students to review their knowledge; a system which may better prepare them for complex application in clinical settings [29]. Indeed, flashcards have been used as a simple prescribing aid that has been shown to improve prescribing confidence [30]. Familiarising students with Anki™ at an early stage and providing the framework for students to experiment with learning may also encourage further usage of the program for other applications. Students can choose to use Anki™ in their clinical years, creating their own flashcards to consolidate applied knowledge.

Responses to the intervention by the participating preclinical student cohort were largely positive, with use of the spaced-repetition scheduled revision being the predominant mode. Student views were largely congruent with the expected benefits of the intervention; particularly of the strength of the intervention as a comprehensive, prepared resource covering their medical pharmacology curriculum. Notably, students perceived themselves engaging with the resource long-term and expressed an interest in similar resources developed for other medical course content, which potentially serves as a more accurate reflection of the overall perception of the resource potential. The prospect for longer-term engagement in the resource is particularly notable as the very nature of Anki™ -based revision essentially ensures re-exposure to, and retention of, curriculum materials well into the future [24, 31]. Based on our findings, the best strategy to implement flashcards in the medical curricula would be introduce them during preclinical years as an adjunct to other learning methods (lectures and tutorials), to not only support the memorisation and understanding of concepts as a prerequisite for higher order learning, but provide a structure for long-term revision.

One particular issue identified by students was a perception of excessive breadth and depth within the covered material, which has been noted in the pharmacology education literature [3]. This may be exacerbated in the case of students that struggle with performing scheduled revision, where reviews may accumulate and result in distress and discouragement. Our view is that any prepared resource such as this should strive to be comprehensive to ensure long-term viability across years of study. However, to address this issue, further support should be offered to students on how to tailor their Anki™ program to their particular needs. Examples could include providing additional flashcard metadata to categorise content based upon academic importance or further instructional material to encourage the personalisation of settings (i.e. limiting daily reviews) and how to omit cards from scheduled reviews [32, 33]. While the number of times students used the flashcard to revise their knowledge was not captured in this study, the Anki™ program has inbuilt statistics that identifies number of reviews, review time, review intervals and percentage of correct reviews, which may be examined in a future study.

The next step in this project is to provide open access publication of these Anki™ resources to other medical schools, particularly to those in countries or regions with students from lower socioeconomic backgrounds, along with accompanying materials, such as guides and tutorials, as well as the investigation of best practices for implementation in a wider context.

This study has several limitations. Low response rates for the primary survey and focus groups may have resulted in a significant bias. Indeed the focus group participants are a subset of those who completed the survey, further reducing participation rates. As students were invited through student noticeboards, a passive modality, a selection bias within our sample of the student cohort captured in the scoping survey and in focus group interviews may have occurred. Additionally, the participation of students from a single medical school may have reduced the external validity of these results. Nevertheless, most findings are consistent with existing literature regarding medical school learning and the use of spaced-repetition as a technique to improve outcomes [13, 27]. The replication of this study in other university cohorts should be conducted to confirm these findings.

## Conclusion

In this study we described an approach to supporting medical pharmacology education in an Australian medical school via the investigation of student learning techniques and, based on this, the design of an Anki™ spaced-repetition flashcard resource. This resource was well received by participating students, particularly with regards to addressing a significant unmet need for time-efficient strategies regarding ongoing revision of medical pharmacology. In this way, we believe that this approach may be broadly applied across the curriculum to improve medical student education.

### Practice points

-
  - Medical students appear to engage minimally with ongoing in-semester revision as a result of fast-paced integrated curricula.
-
  - Evaluations of student cohort learning can lead to significant insight to guide interventions.
-
  - Spaced-repetition flashcard resources are well regarded, well received and attract long term engagement when designed as a curriculum specific, prepared resource on open source and freely available software.
-
  - Spaced-repetition flashcard resources are best implemented during the preclinical phase of the medical curriculum to support knowledge acquisition and retention through structured revision.

## Supplementary Information

**Additional file 1.**

## Data Availability

The datasets generated and/or analysed during the current study are not publicly available due potential breach of privacy and confidentiality of participants but are available from the corresponding author on reasonable request.
