# Supplementary material for: A spaced-repetition approach to enhance medical student learning and engagement in medical pharmacology
Source: BMC Med Educ. 2022 May 2;22:337. doi: 10.1186/s12909-022-03324-8 (PMC9063128; doi:10.1186/s12909-022-03324-8)
Supplement: Supplementary file 1 — Additional file 1. [file 12909_2022_3324_MOESM1_ESM.docx]

**A Spaced-Repetition Approach to Enhance Medical Student Learning and Engagement in Medical Pharmacology**

**Appendix 1: Study Skills**

In this section you are asked questions about the resources, techniques and tools you utilise to study pharmacology. Please respond truthfully, so that your answers will be representative of your current study habits.

1. How long do you spend studying pharmacology per week (______ hours on average, during semester teaching periods)?
   1. How long do you spend learning new pharmacology content per semester week (______ hours on average, during semester teaching periods)?
   2. How long do you spend revising pharmacology content per semester week (______ hours on average, during semester teaching periods)?
   3. How long you spend revising pharmacology content during before an exam (_______ hours on average)

Assign a rating of 1 (Never) to 5 (Very often) for questions 2-5.

1. What prepared **resources** do you use to study pharmacology?
   1. Monash University pharmacology lectures
   2. Monash University prescribed pharmacology textbooks
   3. Non-prescribed pharmacology textbooks
      1. Specify (Type in)
   4. Online resources
      1. AMH
      2. eTG
      3. UpToDate
      4. Wikipedia
      5. Google searches
   5. Other (Type in)
2. What **techniques** do you use to first learn a new topic in pharmacology?
   1. Watch/read pharmacology resources (e.g. lecture slides, lecture recordings, prescribed texts, non-prescribed texts etc)
   2. Participate in tutorial/class activities
   3. Self-created resources
      1. Transcribed notes
      2. Summarised notes
      3. Drug summary table
      4. Concept maps
      5. Flow charts
      6. Diagrams
   4. Question banks (i.e. questions from past tutorials, tests or exams)
      1. Monash University provided
      2. Other (Type in)
   5. Flashcards
      1. Physical flashcards
      2. Physical spaced repetition (e.g. Leitner box)
      3. Online flashcards (e.g. Quizlet)
      4. Spaced repetition (e.g. Anki)
   6. Study groups. If yes, in what way(s)?
      1. For company (i.e. independently working in the group)
      2. For incidental collaboration (i.e. occasional discussion of difficult concepts)
      3. Teaching one another
      4. Taking collaborative notes
         1. Transcribed notes
         2. Summarised notes
         3. Writing flashcards
            1. Physical flashcards
            2. Physical spaced repetition (e.g. Leitner box)
            3. Online flashcards (e.g. Quizlet)
            4. Spaced repetition (e.g. Anki)
      5. Testing one another
      6. Collaborative flashcard sessions
      7. Other (Specify)
   7. Other (Type)
3. What **techniques** do you use to revise pharmacology following classes specifically (i.e. **not** during SWOTVAC)?
   1. Not Applicable
   2. Watch/read pharmacology resources (e.g. lecture slides, lecture recordings, prescribed texts, non-prescribed texts etc)
   3. Reading self-created pharmacology resources again
      1. Transcribed notes
      2. Summarised notes
      3. Drug summary table
      4. Concept maps
      5. Flow charts
      6. Diagrams
   4. Question banks (i.e. questions from past tutorials, tests or exams)
      1. Monash University provided
      2. Other (Type in)
   5. Flashcards
      1. Physical flashcards
      2. Physical spaced repetition (e.g. Leitner box)
      3. Online flashcards (e.g. Quizlet)
      4. Spaced repetition (e.g. Anki)
   6. Study groups. If yes, in what way(s)?
      1. For company (i.e. independently working in the group)
      2. For incidental collaboration (i.e. occasional discussion of difficult concepts)
      3. Teaching one another
      4. Reading collaborative notes
         1. Transcribed notes
         2. Summarised notes
         3. Writing flashcards
            1. Physical flashcards
            2. Physical spaced repetition (e.g. Leitner box)
            3. Online flashcards (e.g. Quizlet)
            4. Spaced repetition (e.g. Anki)
      5. Testing one another
      6. Collaborative flashcard sessions
      7. Other (Type in)
   7. Other (Type in)
4. What **techniques** do you use to revise pharmacology before an exam? (i.e. **during** SWOTVAC)
   1. Not applicable
   2. Watch/read pharmacology resources (e.g. lecture slides, lecture recordings, prescribed texts, non-prescribed texts etc)
   3. Reading self-created pharmacology resources again
      1. Transcribed notes
      2. Summarised notes
      3. Drug summary table
      4. Concept maps
      5. Flow charts
      6. Diagrams
   4. Question banks (i.e. questions from past tutorials, tests or exams)
      1. Monash University provided
      2. Other (Type in)
   5. Flashcards
      1. Physical flashcards
      2. Physical spaced repetition (e.g. Leitner box)
      3. Online flashcards (e.g. Quizlet)
      4. Spaced repetition (e.g. Anki)
   6. Study groups. If yes, in what way(s)?
      1. For company (i.e. independently working in the group)
      2. For incidental collaboration (i.e. occasional discussion of difficult concepts)
      3. Teaching one another
      4. Reading collaborative notes
         1. Transcribed notes
         2. Summarised notes
         3. Writing flashcards
            1. Physical flashcards
            2. Physical spaced repetition (e.g. Leitner box)
            3. Online flashcards (e.g. Quizlet)
            4. Spaced repetition (e.g. Anki)
      5. Testing one another
      6. Collaborative flashcard sessions
      7. Other (Type in)
   7. Other (Type in)
